# Retraction: Does changing from a first generation antipsychotic (perphenazin) to a second generation antipsychotic (risperidone) alter brain activation and motor activity? A case report

**DOI:** 10.1186/1756-0500-6-332

**Published:** 2013-08-20

**Authors:** Jan Øystein Berle, Else-Marie Løberg, Ole Bernt Fasmer

**Affiliations:** 1 Division of Psychiatry, Haukeland University Hospital, Bergen, Norway

## Retraction

The authors have retracted this article [1] as the fMRI data presented in the case report are incorrect. The activation data reported for session 1 are the activation data for session 2 and vice versa. As a result the discussion and conclusions of the case report are based on the wrong set of data and are no longer valid. The authors apologise for the error.
